# Comprehensive analysis of the immune implication of FABP4 in colon adenocarcinoma

**DOI:** 10.1371/journal.pone.0276430

**Published:** 2022-10-20

**Authors:** Dabin Wu, Ling Xiang, Linglong Peng, Haitao Gu, Yunhao Tang, Haoyun Luo, Hang Liu, Yaxu Wang

**Affiliations:** 1 Department of Gastrointestinal Surgery, The Second Affiliated Hospital of Chongqing Medical University, Chongqing, China; 2 Department of Clinical Nutrition, The Second Affiliated Hospital of Chongqing Medical University, Chongqing, China; All India Institute of Medical Sciences, INDIA

## Abstract

**Background:**

Fatty acid-binding protein 4 (FABP4) has been reported to be associated with tumor progress and poor prognosis in various cancers. However, the relationship between FABP4 expression and tumor immunity in colon adenocarcinoma (COAD) is still poorly understood.

**Methods:**

FABP4 mRNA expression was analyzed using The Cancer Genome Atlas (TCGA)-COAD data. FABP4 protein staining was performed by immunohistochemistry (IHC) staining in our 10 paired COAD samples and corresponding adjacent noncancerous tissues. The association between FABP4 and immune cell infiltration was evaluated by Tumor Immune Estimation Resource (TIMER) database. FABP4 coexpressed genes were identified based on Cancer Cell Line Encyclopedia (CCLE) database, which were employed for further enrichment analysis. FABP4 related immunomodulators was identified by Tumor and Immune System Interaction Database (TISIDB) database, and a prognostic risk signature was constructed based on FABP4-related immunomodulators using stepwise Cox regression analysis. A nomogram consists of FABP4 related immunomodulators signature and clinical parameters was developed to predict the overall survival (OS).

**Results:**

In TCGA data, we found that the decreased FABP4 mRNA expression in COAD samples compared with normal samples, and low FABP4 mRNA expression was associated with B cells, CD4+ T cells, CD8+ T cells, myeloid dendritic cells, macrophages, and neutrophils. In our 10 paired samples, the protein levels of COAD were lower in all COAD tissues than in their adjacent noncancerous tissues. Functional enrichment analysis revealed that FABP4 coexpressed genes were mostly enriched in immune-related pathways. Based on 54 FABP4-related immunomodulators, a 2-gene FABP4-related prognostic risk signature was developed, and the signature stratified the patients into the high-risk and low-risk groups with statistically different survival outcomes. The Nomogram consists of the prognostic signature and clinical parameters had a certain predictability for prognosis of COAD patients.

**Conclusion:**

These findings suggest that FABP4 is associated with 2-gene immune signature which also correlate with the prognosis of COAD patients.

## Introduction

Among human malignant tumors, the incidence of colon adenocarcinoma (COAD) ranks third, and the mortality rate ranks second [1]. The poor prognosis of COAD is in part due to delayed colonoscopy and the lack of early detection of special biomarkers for colon cancer. The occurrence and development of COAD is very slow, taking several years or even decades. However, most patients are already in the advanced stage when they are diagnosed with COAD, and their prognosis is poor [2]. Although many treatments have been used to treat COAD, such as surgery, chemotherapy and targeted therapy, the survival rate is still low. It is urgent to identify a valuable evaluation biomarker of therapy effectiveness in COAD patients.

Immune cells are an important part of the tumor microenvironment [3], and immunotherapy provides a new supplement to the traditional treatment [4]. In recent years, immunotherapy has been applied to various tumors, and the efficacy of immunotherapy in colon cancer has also made some progress. In clinical applications, immune checkpoint inhibitor therapy has achieved ideal results for COAD patients with mismatch repair defects and high microsatellite instability [5]. However, patients with other subtypes of COAD have not achieved good treatment effect. Hence, further exploration of the immune-related biomarkers in COAD may provide better treatment effect.

Fatty acid-binding protein 4 (FABP4) is an important molecular member of FABP protein family, and mainly found in adipose tissue and macrophages [6]. It has been well established that FABP4 participated in regulating metabolism and inflammatory response and involved in various metabolic and cardiovascular diseases [7]. Recently, the effects of FABP4 on tumor malignant behavior were also reported, and it may represent a novel biomarker for cancer therapeutic target. For example, Li et al. reported that FABP4 was overexpressed in cervical cancer and FABP4 induce tumor cell invasion through upregulated E-cadherin expression [8]. Masana et al. also reported a cancer-promoting role of FABP4 in breast cancer, in which they suggest tumor cell growth was accelerated when exogenous FABP4 was added into the MCF-7 cells [9]. However, in hepatocellular carcinoma, the expression of FABP4 was downregulated and its expression was negatively related to the patients’ prognosis [10]. In addition, Wu et al. also demonstrated that FABP4 expression was decreased in endometrial cancer tissues, and upregulation of FABP4 suppressed tumor growth and metastasis ability by regulation of PI3K/Akt pathway [11]. Moreover, a previous study showed that FABP4 in endothelial cells is induced by the NOTCH1 signaling pathway, which is involved in the resistance mechanism of antiangiogenic tumor therapy [12]. Taken together, these studies indicate that FABP4 may play an important and dual cancer-promoting or suppressing role in human cancers. However, the potential function of FABP4 in COAD and whether it is related to tumor immune infiltrates remain unclear.

In this study, FABP4 expression and its relationship with immune cells, immune-related pathways and immunomodulators was investigated. Finally, we constructed a prognostic risk signature for prognosis prediction in TCGA-COAD patients based on FABP4-related immunomodulators.

## Materials and methods

### Immunohistochemical staining

The study was approved by the Ethics Committee of the Second Affiliated Hospital of Chongqing Medical University (Chongqing, China, Ethical approval number: (2022) 647). Written informed consent was obtained for experiments with human subjects. Samples from 10 patients diagnosed with COAD and received primary surgery at the Second Affiliated Hospital of Chongqing Medical University were randomly selected for immunohistochemical staining (All samples were collected surgically on March 8–16, 2022). Three pairs of samples (COAD tissues and adjacent noncancerous tissues) were collected from each patient. Before immunohistochemical staining experiment started, all samples were completely de-identified. According to instructions of the reagent manufacturer, FABP4 was analyzed by immunohistochemistry applying formalin-fixed, paraffin-embedded tissue blocks. Briefly, paraffin parts were dewaxed, and endogenous peroxidase activity was blocked with 0.3% hydrogen peroxide for 10 min at corresponding primary antibodies (Abcam, England) overnight at 4°C overnight and then with a secondary antibody at 25°C overnight. The sections were counterstained with hematoxylin. Images were taken with an upright microscope from Leica (Germany) at X100 and X200 magnification, respectively.

### Data acquisition

The mRNA expression data and related clinical information of COAD were downloaded from The Cancer Genome Atlas (TCGA) website ((https://portal.gdc.cancer.gov/). The TCGA-COAD dataset contained 473 cancerous and 41 normal tissues, and cases with insufficient or missing data were excluded. The expression profile of TCGA-COAD datasets was processed by R package “limma”.

### Analysis of the relationship between FABP4 expression and infiltration immune cell

Tumor Immune Estimation Resource (TIMER) is a website tool that can comprehensively analyze the infiltration immune cells of tumor tissues (http://timer.cistrome.org/). This database contained four immune-related analysis modules that was used to obtain immune related information of targeted gene, gene mutation, copy number alteration and prognosis [13]. In this study, we used this tool to explore the relationship between infiltration immune cells and FABP4 expression levels. Single Cell Portal, a database containing multiple single cell sequencing data, was used to analyze the relationship between FABP4 expression and immune cells in COAD in this study (https://singlecell.broadinstitute.org/single_cell). Data on single cells were obtained from the Study: Human Colon Cancer Atlas (c295).

### GO and KEGG enrichment analysis

Cancer Cell Line Encyclopedia (CCLE) database is an open tumor-related research database for tumor cell lines. In this study, the expression profile of COAD cell lines was extracted from CCLE dataset. The coexpressed genes of FABP4 were identified in CCLE dataset using the “limma” R package with correlation coefficient> 0.5 and p-value< 0.05 [14]. Gene Ontology (GO) is a project created to describe the function of gene products. It includes three parts: the activity at the molecular level of the gene product, the location of the cell structure, and the completed biological process. Kyoto Encyclopedia of Genes and Genomes (KEGG) is a database that integrates genome, chemistry and system function information. In this study, we used GO and KEGG enrichment analysis method to explore the signaling pathways of FABP4 coexpressed genes.

### Analysis of FABP4-associated immunomodulators

Tumor and Immune System Interaction Database (TISIDB) is a database containing a large amount of tumor-immune interaction information, and we obtained FABP4-associated immunomodulators from this database (http://cis.hku.hk/TISIDB/) [15]. The Protein–Protein Interaction (PPI) network is helpful for us to study the mechanisms of diseases and the targets of new drugs, and it helps us to better understand the interactions between the end products of genes and proteins. Using the Search Tool for the Retrieval of Interacting Genes (STRING) database, we constructed a PPI network of FABP4-related immunomodulators (http://string-db.org/). Furthermore, based on FABP4-related immunomodulators, we performed a functional enrichment analysis by WebGestalt online tool (http://www.webgestalt.org/) [16].

### Prognostic analysis

Based on FABP4-associated immunomodulators, we construct a prognostic risk model to predict the survival of TCGA-COAD patients and used the Cox regression model to conduct stepwise variable selection [17]. The expression level of each prognostic gene and their corresponding coefficient can be obtained in the Cox regression risk model. In this model, the risk score of each patient can be counted with the following formula: risk score = a_1_x_1_+ a_2_x_2_+ … + a_i_x_i_. Here, a_i_ represents the coefficient of each gene, and x_i_ represents the expression level of each gene. Kaplan–Meier curves was used to explore the relationship between risk score and patient overall survival. Using a time-dependent receiver operating characteristic (ROC) curve, the effect and application value of risk scores were evaluated [18]. The prognostic value of risk score, age, gender, stage was assessed by univariate and multivariate Cox regression analyses. Then, a prognostic nomogram was constructed using R package “rms”, in which the scoring standard was based on the regression coefficients of all variables and the survival probability for each patient was visualized according to the cumulative points of all variables. The calibration curve was applied to evaluate the prediction effect of the nomogram.

### Statistics

Our statistical analysis was performed using R version 4.0.5. Student’s t test was used to determine the FABP4 expression differences between COAD tissues and normal tissues. Univariate and multivariate Cox regression analyses were used to construct prognostic signature. P<0.05 was considered statistically significant.

## Results

### Analysis between FABP4 expression and infiltration of immune cell in COAD

**Fig 1:**
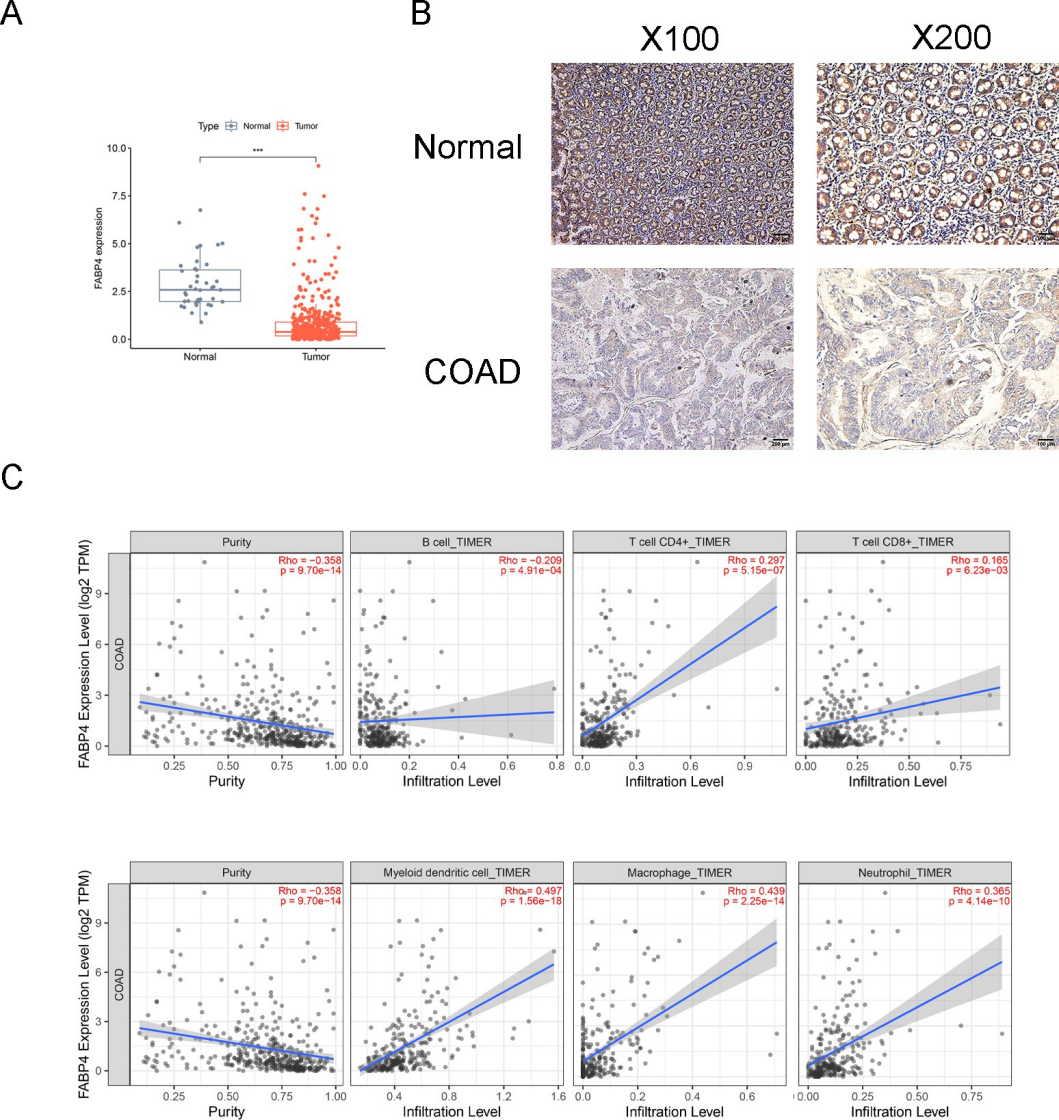
FABP4 expression correlated with infiltration immune cells in COAD patients. (A) The mRNA expression levels of FABP4 in TCGA-COAD samples and normal samples, *** p <0.001. (B) The protein level of FABP4 in COAD tissues and adjacent noncancerous colon tissues (magnified, X100 and X200). (C) Correlation analysis of FABP4 expression and immune infiltration cells based on TIMER database.

The mRNA expression levels of FABP4 in TCGA-COAD tissues (n = 473) were significantly lower than those in normal tissues (n = 41) (Fig 1A, p <0.001). Furthermore, our immunohistochemical results confirmed that the protein level of FABP4 is downregulated in COAD tissues compared to adjacent noncancerous colon tissues (Fig 1B). Moreover, we used the TIMER database to further understand the correlation between FABP4 expression and infiltrating immune cells in COAD patients. As shown in Fig 1C, FABP4 expression was significantly associated with B cells, CD4+ T cells, CD8+ T cells, myeloid dendritic cells, macrophages, and neutrophils. Our single cell analysis found that FABP4 is mainly expressed in Stromal cells and Myeloid cells (S1 Fig). These results suggest that FABP4 play an important role in tumor immune cell infiltration of COAD patients.

### Functional enrichment analysis of FABP4 coexpressed genes

**Fig 2:**
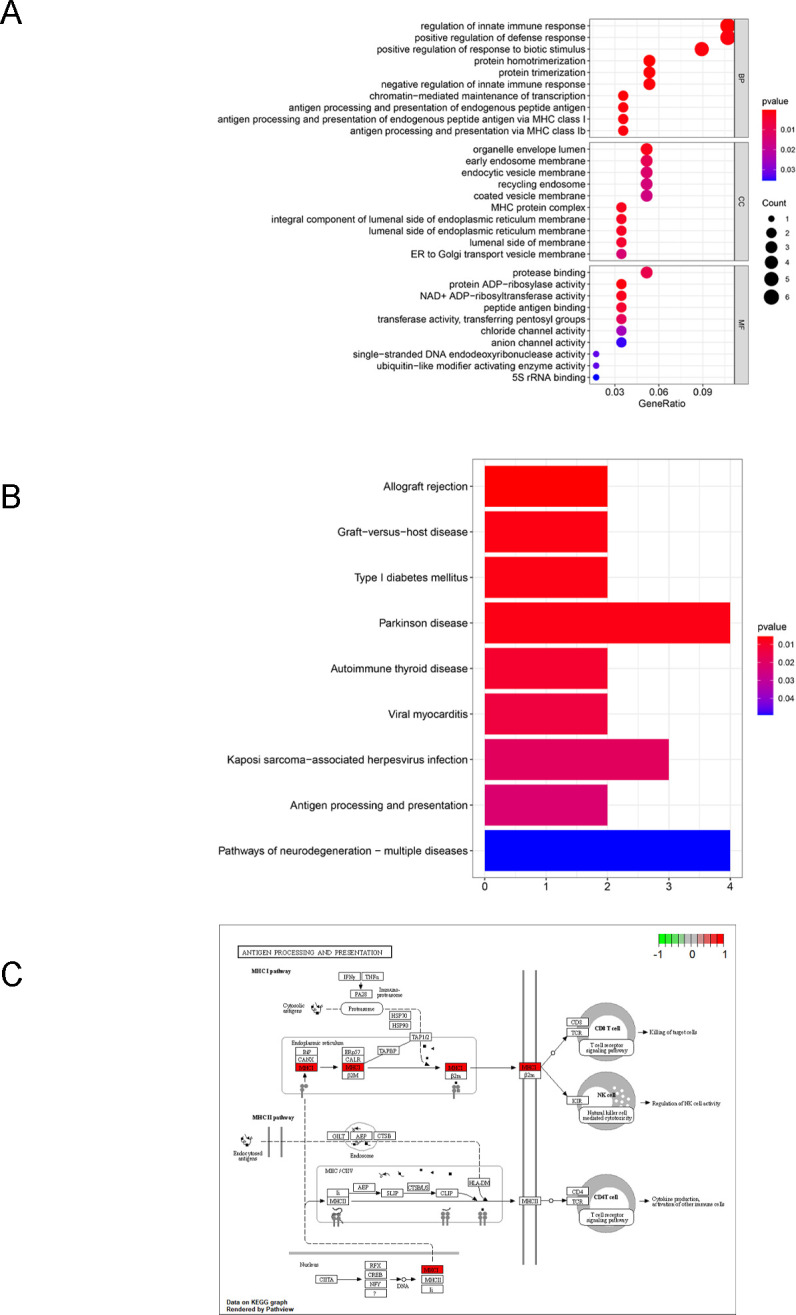
Functional enrichment analyses of FABP4 coexpressed genes in COAD. (A) GO and (B) KEGG analysis of the coexpressed genes in COAD. (C) FABP4 related coexpression genes was enriched in antigen processing and presentation pathway.

To explore the biological function of FABP4 in COAD, we performed a coexpression analysis based on FABP4 expression using the expression profile of CCLE database. Then, these coexpressed genes were chosen to conduct GO and KEGG analyses. The annotation function of GO analysis was shown in Fig 2A. KEGG results showed that the coexpressed genes related to FABP4 were significantly enriched in the immune-related pathways, such as innate immune response, defense response, and antigen processing and presentation (Fig 2B). Specially, those activated molecules in the antigen processing and presentation pathway based on FABP4 related coexpression genes was shown in Fig 2C.

### Potential immune modulatory function of FABP4 in COAD

**Fig 3:**
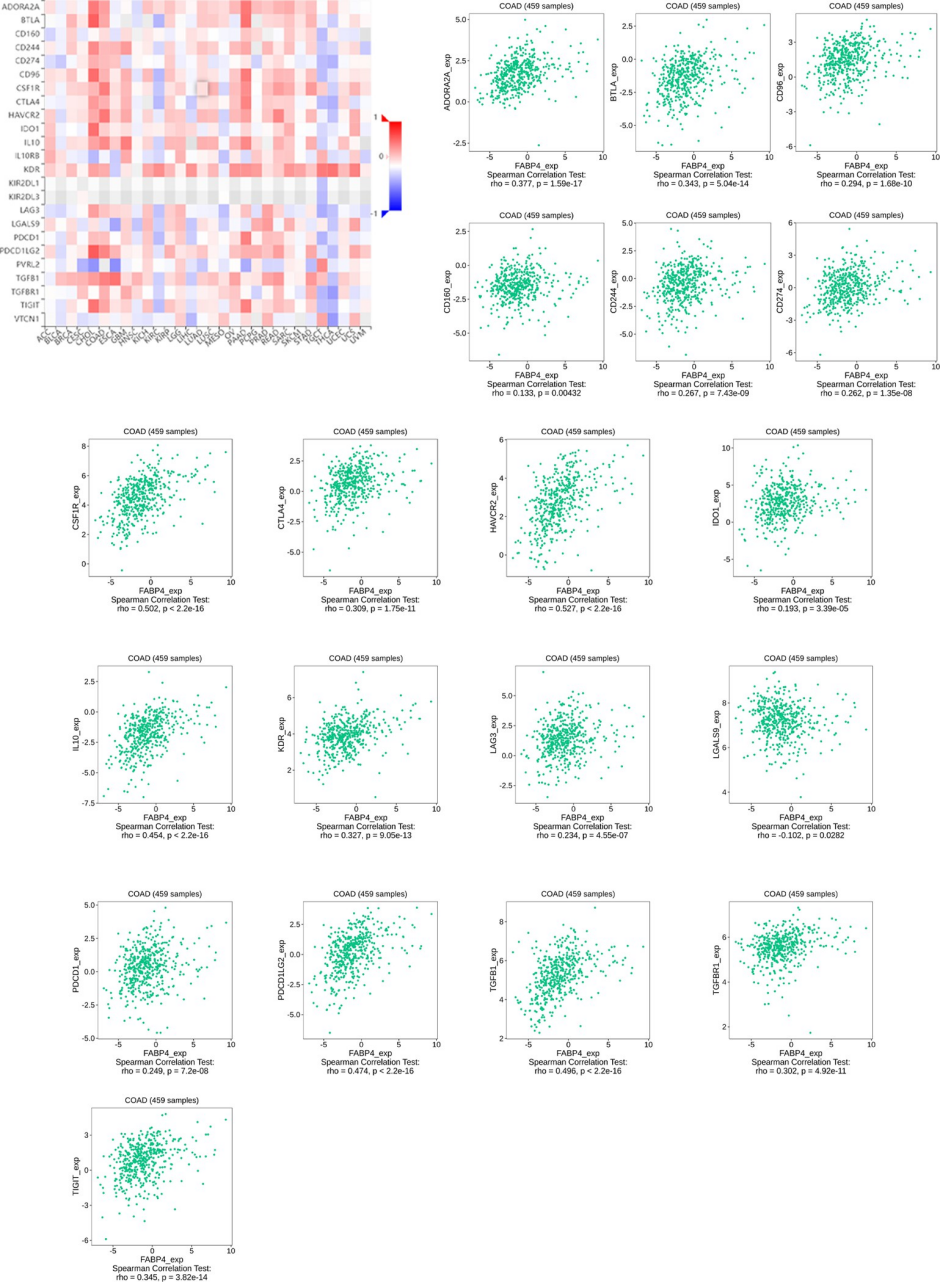
Immunoinhibitors associated with the FABP4 gene.

**Fig 4:**
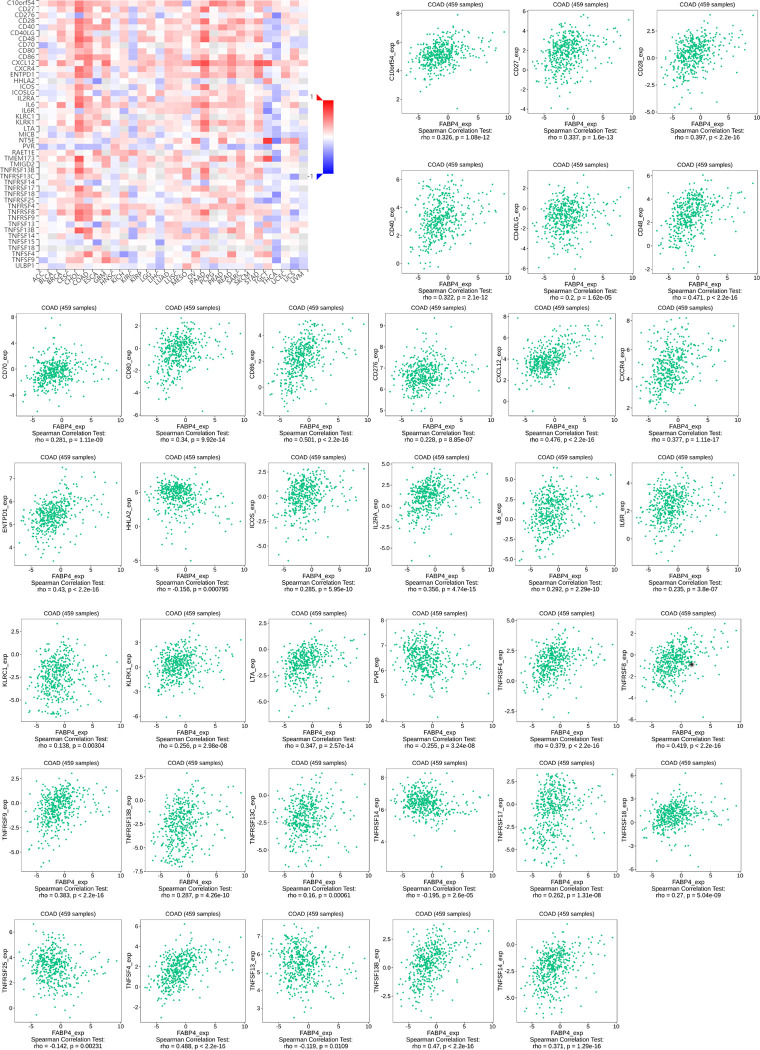
Immunostimulators associated with the FABP4 gene.

**Fig 5:**
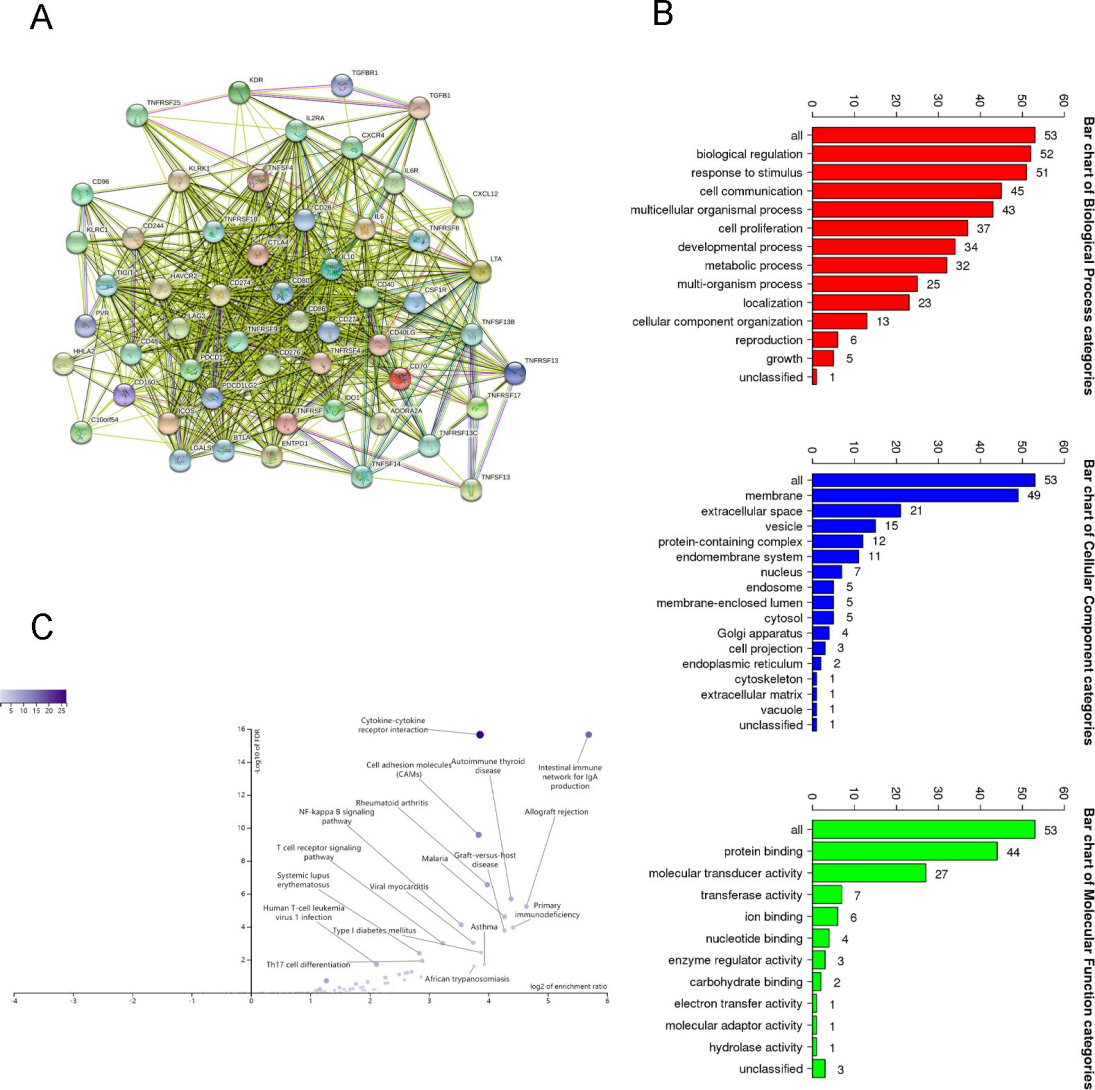
Functional analysis of FABP4-related immunomodulators. (A) PPI network of FABP4-associated immunomodulators in COAD. (B) GO annotation of 54 FABP4-associated immunomodulators in COAD. (C) KEGG pathway analysis of 54 FABP4-associated immunomodulators.

The role of immunomodulators is to regulate the immune response in immunotherapy, change the immune response state and improve the corresponding immune function. We further identified 19 immunoinhibitors (ADORA2A, BTLA, CD96. CD160, CD244, CD274, CSF1R, CTLA4, HAVCR2, IDO1, IL10, KDR, LAG3, LGALS9, PDCD1, PDCD1LG2, TGFB1, TGFBR1, and TIGIT) (Fig 3) and 35 immunostimulators (C10orf54, CD27, CD28, CD40, CD40LG, CD48, CD70, CD80, CD86, CD276, CXCL12, CXCR4, ENTPD1, HHLA2, ICOS, IL2RA, IL6, IL6R, KLRC1, KLRK1, LTA, PVR, TNFRSF4, TNFRSF8, TNFRSF9, TNFRSF13B, TNFRSF13C, TNFRSF14, TNFRSF17, TNFRSF18, TNFRSF25, TNFSF4, TNFSF13, TNFSF13B, and TNFSF14) (Fig 4) that were closely correlated with FABP4 expression in COAD. Next, we used STRING tool to construct a PPI network based on the 54 FABP4-related immunomodulators (Fig 5A). The potential function of the 54 immunomodulators was annotated with GO analysis and the result was shown in Fig 5B. Then, KEGG pathway enrichment analyses showed that of the 54 FABP4-related immunomodulators were involved in immune processes including T cell receptor signaling pathway (Fig 5C).

### The prognostic value of FABP4-associated immunomodulators in COAD

**Fig 6:**
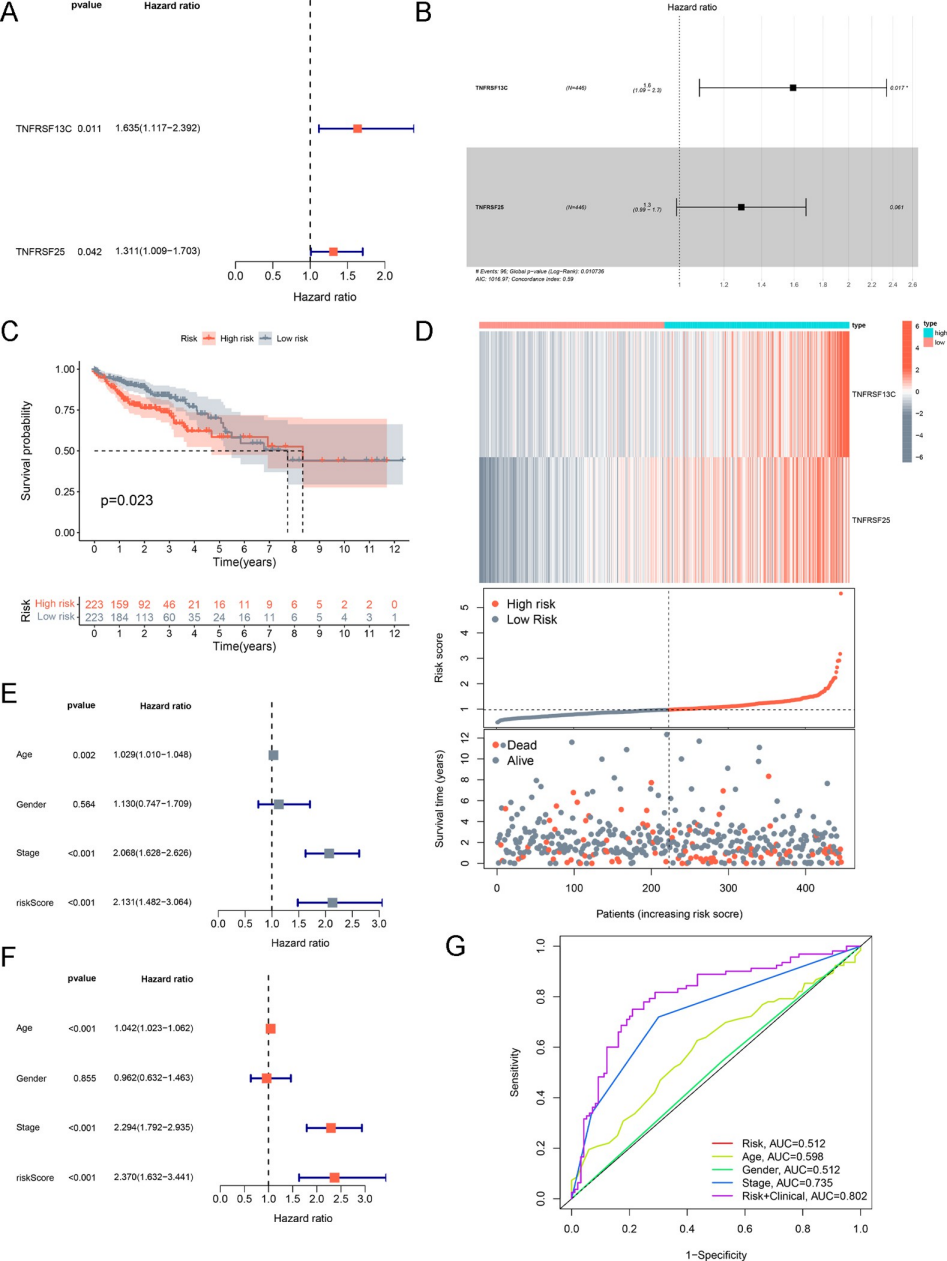
Development of prognostic gene signatures based on 54 FABP4-associated immunomodulators. (A) Univariate Cox analysis of the 54 immunomodulators associated with overall survival in TCGA-COAD patients. (B) Multivariate Cox analysis for constructing a 2-immunomodulators prognostic signature. The Hazard radio was shown in the plot. (C) Kaplan–Meier curves of the overall survival between high-risk and low-risk groups based on the 2-immunomodulators prognostic signature. (D) The gene expression profiles, and survival statuses related to risk scores in TCGA-COAD patients. (E) Univariate and (F) multivariate Cox regression analyses of the risk score in COAD. (G) The ROC curves at 3-years for COAD.

To further investigate the prognostic value of FABP4-associated immunomodulators in COAD, a stepwise Cox regression analysis was performed. Univariate Cox regression analysis identified TNFRSF13C and TNFRSF25 immunomodulators that were associated with patient overall survival in TCGA-COAD dataset (Fig 6A). Intriguingly, following multivariate analysis, a two-gene prognostic signature was constructed based on the formula mentioned in our methods (Fig 6B). In this prognostic signature, the risk score of each patient was obtained and patients were divided into high-risk groups and low-risk groups according to their risk score. The results of survival analysis elucidated that patient with high-risk scores had a poorer prognosis (Fig 6C, P = 0.023). The distribution of risk score, patient survival status and the-risky two gene expression profile were visually shown in Fig 6D. Furthermore, our results showed that the risk score was an independent prognostic factor of COAD patients using univariate and multivariate Cox regression analyses (Fig 6E and 6F). The receiver operating characteristic (ROC) curve revealed that the area under the curve (AUC) value was 0.802 after the risk score and clinical information were combined (Fig 6G).

### Prognostic nomogram

**Fig 7:**
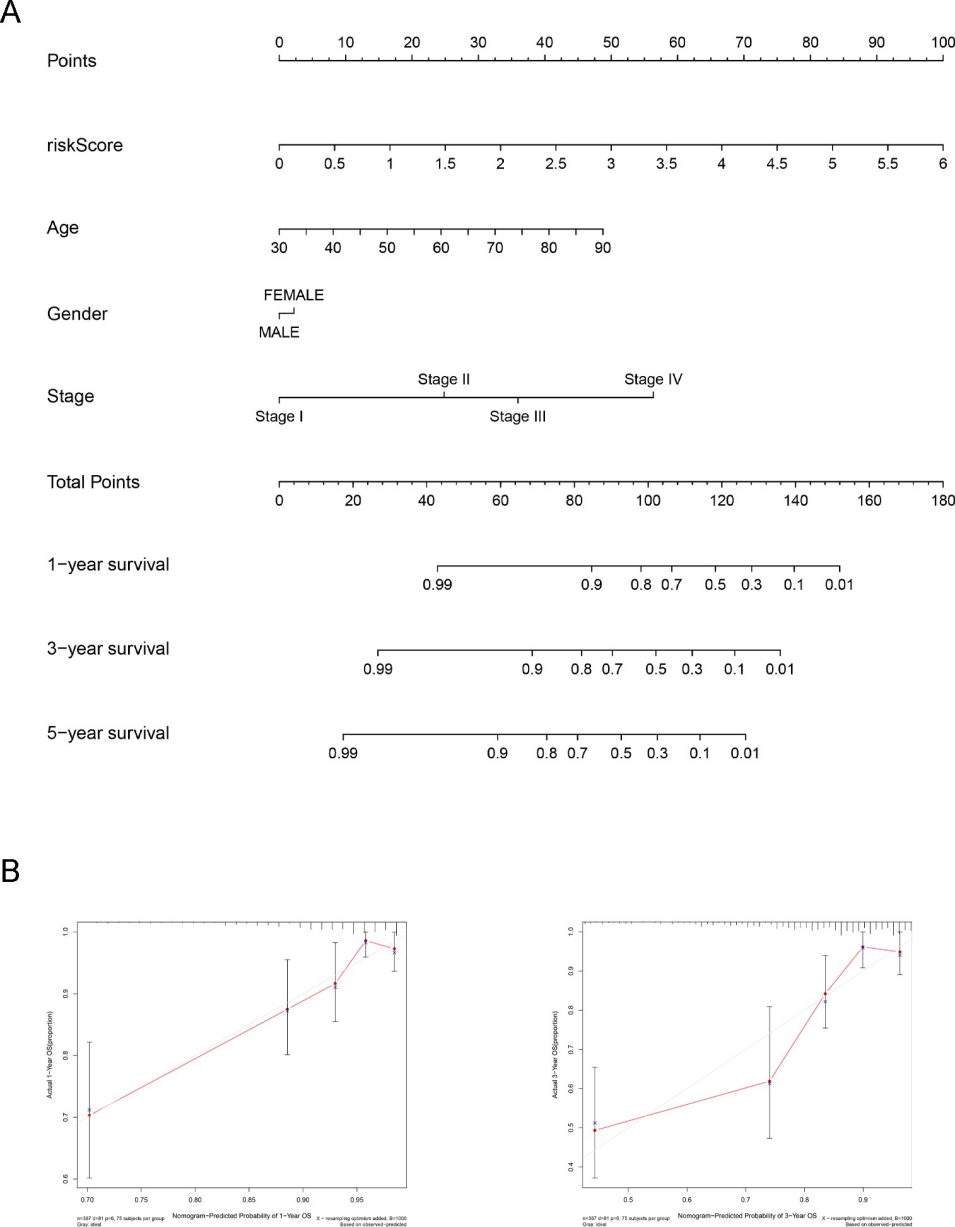
Prognostic nomogram of TCGA-COAD patients. (A) Nomogram for predicting the 1-, 3-, and 5-year overall survival of TCGA-COAD patients. (B) The calibration curve of 1- and 3-year overall survival of TCGA-COAD patients.

Finally, we constructed a nomogram to visualize the impact of variable factors such as risk score, stage, age, and sex on the prognosis of TCGA-COAD patients (Fig 7A). Our prognostic nomogram reached a C-index of 0.584. We tried to calibrate the nomogram to evaluate the validity and reliability of the model. The calibration curve depicts the relationship between the actual occurrence rate and the predicted occurrence rate. Our results revealed that the nomogram had good performance in predicting the 1-year and 3-year survival of COAD patients (Fig 7B).

## Discussion

The pathogenesis and treatment of COAD are the important subjects of cancer biology research. Recent advances in testing and drugs have greatly improved the survival rate of colon cancer [19]. Specially, due to the research progress of immune checkpoint inhibitors, immunotherapy has widely applied to the treatment of COAD patients [20]. However, only small part of patients can be benefited from immunotherapy, and the mortality of advanced patients are still uncontrollable. In this study, we found a close relationship between tumor immunity and FABP4 expression in COAD patients, which may be a useful biomarker for the immunotherapy of colon cancer.

FAPB4 is a tumor suppressor, the expression of FABP4 is significantly correlated with tumor stage, disease-free survival (DFS) and overall survival (OS) [21]. Especially, overexpression of FABP4 in tumor-associated macrophages promotes breast cancer growth [22], and blocking FABP4 expression in endothelial cells reduces angiogenic activity and tumor growth [21]. In our study, FABP4 expression is also downregulated in TCGA-COAD cohort, and immunohistochemical results also confirmed that the protein level of FABP4 is downregulated in COAD tissues. Intriguingly, based on the TIMER analysis tool, our results showed a close correlation between FABP4 expression and the abundances of B cells, CD4+ T cells, CD8+ T cells, myeloid dendritic cells, macrophages, and neutrophils, indicating that FABP4 expression affected infiltration immune cells in COAD. Taken together, these results suggest that the expression of FAPB4 can be used as an immune-related biomarker.

In this study, we performed a coexpression analysis based on FABP4 expression using the colon cell lines data from CCLE database. Next, GO and KEGG analyses were conducted based on these coexpressed genes. The results showed that the coexpressed genes related to FABP4 were enriched in immune-related pathways, such as innate immune response, defense response, and antigen processing and presentation. The innate immune response exert antitumor effects at different stages of tumorigenesis due to the plasticity of the immune microenvironment [23]. Moreover, the natural processing and presentation of antigens involves loading peptides onto major histocompatibility complex (MHC) class I molecules [24]. Tumor antigens are displayed on the cell surface through MHC class I antigens in the antigen processing and presentation process [25]. To produce an effective antitumor response, tumor antigens must be taken up by dendritic cells (DCs) and presented for CD8+ T cell activation [25]. This is in line with our result that FABP4 expression was positively correlated with CD8+ T cells and DCs.

Previous studies have shown that immune-related signature can predict the prognosis of patients with various tumors. Wen et al. constructed an immune risk signature containing 10 immune-related genes to predict the prognosis of colon cancer patients [26]. Li et al. reported an immune signature based on 2414 lung cancer patients, which was reliable and useful to assess the overall survival of patients as it was validated by three independent cohorts [27]. Zhuang et al. developed a 4-genes M1 macrophages prognostic signature in thyroid cancer based on a special immune cell subset [28]. In addition, based on the immune score, Liu et al. constructed a 25-gene prognostic signature to predict the prognosis of breast cancer [29]. In this study, based on FABP4-associated immunomodulators, we constructed a 2-immunomodulators signatures to predict the prognosis of COAD. Moreover, we used ROC curves to assess the predictive efficacy of immune signatures. Finally, using Cox regression analyses, we tried to construct a nomogram to predict the prognosis of COAD patients. Our results can provide clinicians with more information about the immune and prognostic role of FABP4 expression in COAD.

Our study has several limits. First, the more in vitro validation experiment was needed. Second, the mechanism of FABP4-mediated immunity in COAD and the prognostic value of the immune signature proposed in our study need to be further explored. We will systematic investigate the immune-related effect and mechanism through experiments and other methods in follow-up research.

In summary, FABP4 expression affects immune cell infiltration, immune-related pathways and immunomodulators. The prognostic signature constructed by FABP4-related immunomodulators is a good predictor for COAD prognosis.

## Supporting information

S1 Fig Single cell analysis of the association of FABP4 with immune cells. Epi: Epithelial cells. TNKILC: T cells, NK cells and ILC cells.
(TIF)
