# Supplementary figures and images for: Comprehensive analysis of the immune implication of FABP4 in colon adenocarcinoma
Source: PLoS One. 2022 Oct 20;17(10):e0276430. doi: 10.1371/journal.pone.0276430 (PMC9584364; doi:10.1371/journal.pone.0276430)

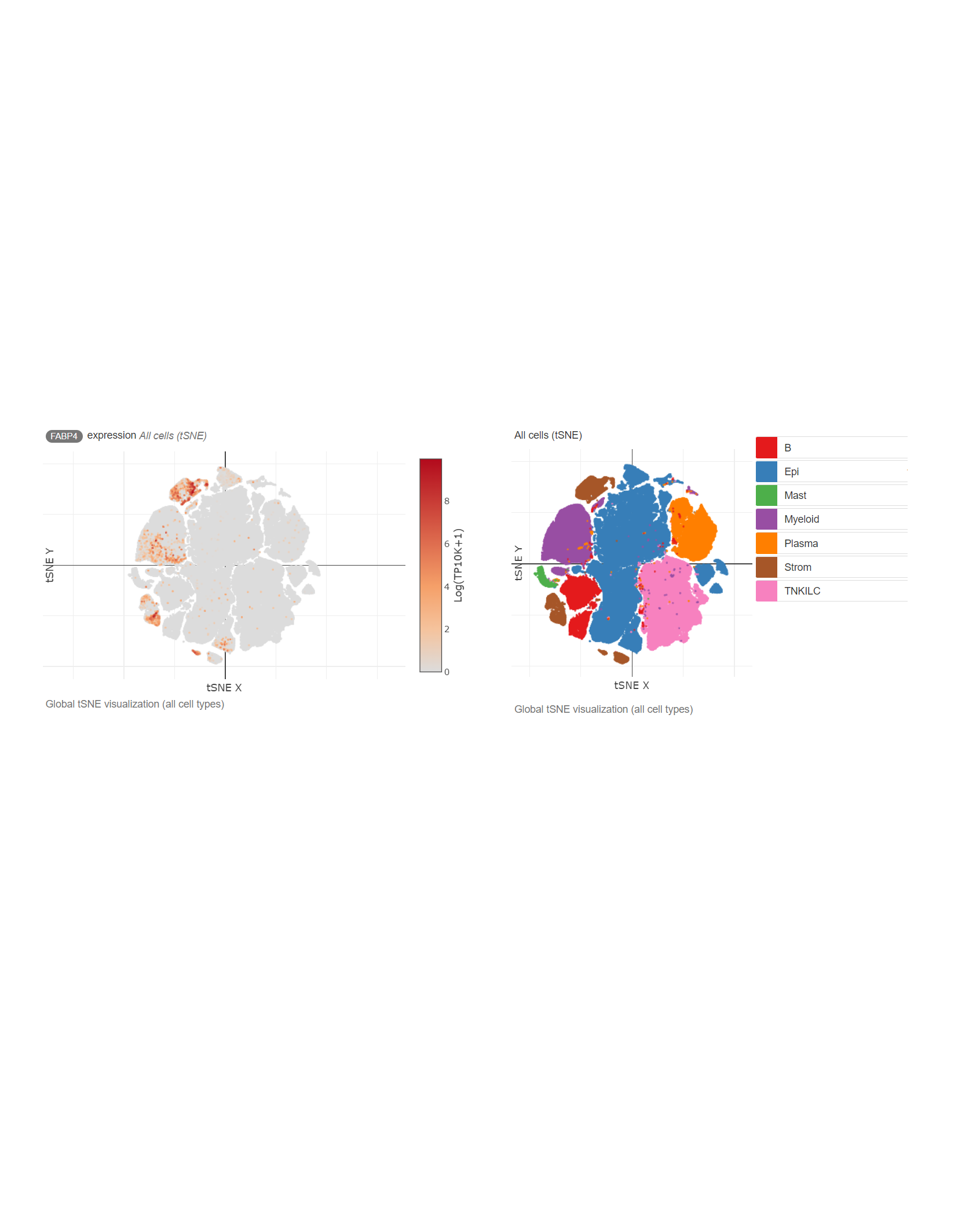

Supplement: S1 Fig — Epi: Epithelial cells. TNKILC: T cells, NK cells and ILC cells. (TIF) [file pone.0276430.s001.tif]
